# Neue Empfehlungen zur palliativen Sedierung

**DOI:** 10.1007/s00482-024-00825-x

**Published:** 2024-09-12

**Authors:** Séverine Marie Surges, Holger Brunsch, Marta Przyborek, Birgit Jaspers, Lukas Radbruch

**Affiliations:** 1 Klinik für Palliativmedizin, Universitätsklinikum Bonn, Venusberg-Campus 1, 53127 Bonn, Deutschland; 2 Bonner Netzwerk für Versorgungsforschung, Universität Bonn, Bonn, Deutschland; 3 Klinik für Palliativmedizin, Universitätsmedizin Göttingen, Göttingen, Deutschland; 4 Task Force on Palliative Sedation, European Association for Palliative Care, Brüssel, Belgien; 5 Helios Klinikum Bonn/Rhein-Sieg, Bonn, Deutschland

**Keywords:** Palliative Sedierung/Entscheidungsfindung, Refraktäres Leid, Palliative Sedierung/Proportionalität, Informierte Einwilligung, Midazolam, Palliative sedation/decision-making, Refractory suffering, Palliative sedation/proportionality, Informed consent, Midazolam

## Abstract

Palliative Sedierung zielt auf die Linderung von refraktärem Leid bei Patienten mit lebenslimitierender Erkrankung ab. Das Rahmenwerk zur palliativen Sedierung der Europäischen Gesellschaft für Palliativmedizin (European Association for Palliative Care [EAPC]) von 2009 wurde vor Kurzem aktualisiert. In Deutschland wurden von der SedPall-Forschungsgruppe ebenfalls Empfehlungen formuliert. Der vorliegende Beitrag beschreibt die soziale und ethische Komplexität der Entscheidungsfindung und fasst die Empfehlungen zusammen. Die Autonomie des Patienten wird hervorgehoben. Die Bestimmung der Refraktärität des Leids soll gemeinsam von Arzt und Patient vorgenommen werden. Die Sedierung soll proportional erfolgen, das heißt, ihre Form und Dauer werden an die individuelle Situation des Patienten angepasst. Die Entscheidungen zur palliativen Sedierung und zur Hydratation sollen getrennt erfolgen. Midazolam gilt als Medikament der ersten Wahl. Besondere Aufmerksamkeit ist den Angehörigen und dem Behandlungsteam zu widmen.

## Abstract (en)

Palliative sedation aims to relieve refractory suffering in patients with life-limiting disease. The 2009 framework on palliative sedation of the European Association for Palliative Care (EAPC) has recently been updated. Recommendations have also been formulated by the research group SedPall in Germany. This article describes the social and ethical complexity of decision-making and summarises the recommendations. Patient autonomy is emphasised. Refractoriness of the suffering should be determined jointly by physician and patient. Sedation should be proportional, that is to say, its form and duration should be adapted to the patient’s individual situation. The decision on palliative sedation and that on hydration involve two separate decision-making processes. Midazolam should be used as first choice. Particular attention should be paid to the patient’s relatives/significant others and the treating team.

## Lernziele

Nach Lektüre dieses Beitrags

- kennen Sie die Definition von palliativer Sedierung und können unterschiedliche Formen der palliativen Sedierung unterscheiden.
- wissen Sie, welche ethischen Fragen im Rahmen der palliativen Sedierung diskutiert werden.
- wissen Sie, wie der Entscheidungsprozess zur Durchführung einer palliativen Sedierung gestaltet werden sollte.
- kennen Sie den empfohlenen stufenweisen pharmakologischen Ansatz zur Durchführung der palliativen Sedierung.
- sind Ihnen Kriterien zum Monitoring während der palliativen Sedierung vertraut.

## Einleitung

Menschen sind am Ende ihres Lebens häufig mit körperlicher, psychischer, sozialer und existenzieller Not konfrontiert [1]. Die Aufgabe der Palliativmedizin besteht in der umfassenden Versorgung dieser Menschen; Ziel ist die Verbesserung der **Lebensqualität**

Lebensqualität

sowohl der Patienten als auch ihrer An- und Zugehörigen [2]. Trotz bestmöglicher palliativmedizinischer Versorgung liegt bei einigen Patienten schweres körperliches, psychisches oder existenzielles Leid vor, bei dem konventionelle Behandlungsoptionen versagen. In diesen Fällen **refraktären Leids**

Refraktäres Leid

kann die gezielte Reduzierung des **Bewusstseins**

Bewusstsein

(palliative Sedierung [PS]) angezeigt sein. Die Europäische Gesellschaft für Palliativmedizin (European Association for Palliative Care [EAPC]) veröffentlichte 2009 ein **Rahmenwerk**

Rahmenwerk

zur Information und zur Entwicklung lokaler Verfahrensrichtlinien für PS, das im Rahmen des europäischen Forschungsprojekts PallSed (https://www.palliativesedation.eu) 2023 aktualisiert wurde [3, 4] Nahezu parallel zu diesem Forschungsprojekt führte die Forschungsgruppe von Ostgathe et al. (https://www.palliativmedizin.uk-erlangen.de/forschung) in Deutschland ein Projekt auf nationaler Ebene zum Thema „Von der Anxiolyse zur tiefen und kontinuierlichen Sedierung“ durch und veröffentlichte hierzu Empfehlungen [5]. Beide Studien folgten einem Mixed-methods-Ansatz. Die aus ihnen resultierenden Empfehlungen sind jeweils in Delphi-Verfahren mit Experten konsentiert worden. Für die EAPC-Empfehlungen wurden insgesamt 91 Teilnehmer aus 28 Ländern in den Konsensprozess eingebunden. Mehrere systematische Literaturübersichten wurden zeitgleich erstellt, deren Ergebnisse in die Formulierung der Empfehlungen eingeflossen sind [6, 7, 8, 9]. Ziel des vorliegenden Beitrags ist es, diese Empfehlungen zusammenzufassen.

### Fallbeispiel

Frau S. (81 Jahre) hat ein metastasiertes Mammakarzinom und leidet an Ataxie, Dysarthrophonie, Intentionstremor mit Schwindel und Übelkeit infolge einer paraneoplastischen zerebellären Degeneration. Ihre Lebenserwartung wird auf sechs Monate geschätzt. Nach sieben erfolglosen Plasmapheresen wurde ihr Zustand als irreversibel eingestuft. Sie ist aufgrund des Schwindels bettlägerig. Trotz Gabe von Dexamethason und Antiemetika löst die geringste Bewegung bei ihr Übelkeit und Erbrechen aus. Die Patientin zeigt keine Anzeichen einer Depression und ihre Entscheidungsfähigkeit ist nicht beeinträchtigt. Sie äußert vehement und wiederholt den Wunsch zu sterben und trifft die Entscheidung, mit sofortiger Wirkung freiwillig auf Essen und Trinken zu verzichten. Sie wünscht dazu eine palliative Sedierung (PS), da die Situation für sie unerträglich sei. Ihre Kinder sind anwesend und unterstützen sie in ihrer Entscheidung.

In einer multiprofessionellen Fallbesprechung, an der drei Ärzte (1 ♂, 2 ♀), vier Pflegefachkräfte (4 ♀), ein Psychoonkologe, eine Sozialarbeiterin und ein Ethiker teilnehmen, werden ein Cluster refraktärer körperlicher Symptome und ein Zustand existenziellen Leids als Indikation für eine PS bewertet. Die Lebenserwartung der Patientin, die aufgrund des bereits begonnenen Verzichts auf Flüssigkeitszufuhr erheblich verkürzt ist, spielt bei der Entscheidung über die Indikation zur PS keine Rolle, wohl aber bei der Wahl der Form der Sedierung.

Mit der Patientin wird eine 24-stündige intermittierende PS vereinbart, anschließend soll die Situation neu bewertet werden. Die PS wird am nächsten Tag mit 1 mg/h Midazolam und einem anfänglichen Bolus von 2 mg begonnen. Zwei weitere Boli von 2 mg werden 30 min und 3 h nach Beginn der PS verabreicht und die Erhaltungsdosis wird bei der Verabreichung des letzten Bolus auf 2,5 mg/h erhöht. Mit dieser Dosis wird ein ausreichendes Maß an Komfort erreicht, weitere Boli sind nicht erforderlich. Nach 24 h wird die PS pausiert, aber auf Wunsch der Patientin dann wieder fortgesetzt. In der Folge verschlechtert sich ihr Zustand rapide und sie verstirbt zwei Tage später.

## Definition

Die PS wird in den neuen Empfehlungen der EAPC definiert „als eine Sedierung, die auf Linderung von refraktärem Leid durch die überwachte proportionale Gabe von Medikamenten zur Bewusstseinsreduktion bei Patienten mit lebenslimitierenden Erkrankungen zielt. Sie hat soziale und ethische Implikationen, die besondere Berücksichtigung bei Patienten, An- und Zugehörigen sowie Behandelnden erfordern“ [4].

Die SedPall-Gruppe verwendet den Begriff der „gezielten Sedierung“ anstelle von „palliativer Sedierung“. Die **gezielte Sedierung**

Gezielte Sedierung

wird als eine Sedierung definiert, die die Folge einer medizinischen Intervention ist und nicht eine Nebenwirkung eines Medikaments oder Teil des natürlichen Sterbeprozesses. Die Fragen der guten Praxis, beispielsweise Indikation und Voraussetzungen für die Sedierung, wurden aus der Grundterminologie ausgeklammert, damit die Definition dieser Praxis nicht von einer Institution zur anderen oder von einem Land zum anderen aufgrund von unterschiedlichen Regeln der guten Praxis variiert [10].

## Indikation: das Prinzip der Refraktärität des Leids

Eine der Voraussetzungen für die Indikationsstellung einer PS ist das Vorliegen refraktären Leids. Unter refraktärem Leid versteht man ein Leid, das vom Patienten als unerträglich empfunden wird und das mit konventionellen Methoden nicht behandelt werden kann, weil diese bereits ausgeschöpft wurden, die unerwünschten Wirkungen zu stark sind oder der Zeitraum bis zur Erreichung der Symptomkontrolle unzumutbar lang wäre.

Die in der Literatur am häufigsten genannten körperlichen Symptome sind Delirium, Atemnot und Schmerzen, aber auch Notfallsituationen in der Sterbephase wie massive Blutungen, Asphyxie, schwere terminale Dyspnoe oder überwältigende Schmerzattacken [6]. Arantzamendi et al. [6] zeigen in ihrer Übersichtsarbeit, dass in 60–90 % der beschriebenen Fälle die Indikation nicht ein Symptom betraf, sondern ein Cluster von ein bis drei Symptomen, deren Kombination zu einer Situation führte, die der Patient als unerträglich empfand. In 10–48 % der Fälle gehörte zu diesem **Symptomcluster**

Symptomcluster

auch psychisches und/oder **existenzielles Leid**

Existenzielles Leid.

Gabl et al. [11] beschreiben existenzielles Leid als „Oberbegriff für die gesamte Erfahrung des Leids, das durch den Verlust der Verbindung zu den Werten, die dem Leben zugrunde liegen, verursacht wird“, und unterteilen dieses in zwei Kategorien: „(1) existenziellen ‚distress‘, der das Elend von Patienten und Angehörigen, die existenziell leiden, beschreibt, während sie noch einen Sinn im Leben sehen, und (2) existenzielle Verzweiflung, die das unerträgliche Leid an einer erfahrenen Kombination aus Sinnlosigkeit und Hoffnungslosigkeit beschreibt, in der ein existenzielles Vakuum zu spüren ist“. Nur in seltenen Fällen wurde psychisches und/oder existenzielles Leid als einzige Indikation für eine Sedierung angegeben [6].

Cassell [12] vertritt die Auffassung, dass eine Person, die leidet, immer in allen Dimensionen betroffen ist, die ihr Wesen ausmachen (physisch, psychisch, sozial, existenziell, spirituell). Daher empfiehlt es sich beim Assessment, nicht nur die Symptome selbst, sondern auch ihre psychischen und sogar existenziellen Auswirkungen zu berücksichtigen. Der Begriff Leid wurde in den neuen Empfehlungen der EAPC bewusst verwendet, um diese holistische Herangehensweise zu reflektieren und dabei nicht nur die physischen und psychologischen Symptome, sondern auch das existenzielle Leid zu berücksichtigen [4]. Bozzaro et al. [13] weisen jedoch auf die Herausforderungen hin, die dieses Konzept des Leids im Rahmen des Entscheidungsprozesses für eine PS mit sich bringe, da die Beurteilung des refraktären Charakters existenziellen Leids weitaus komplexer sei. Dies liegt an seiner **fluktuierenden Natur**

Fluktuierende Natur

und an der Tatsache, dass es durch medizinische oder psychosoziale Maßnahmen, durch die Resilienz des Patienten, aber auch durch das Mitgefühl und die Fürsorge von Angehörigen und Betreuern gelindert werden kann [5]. Gabl et al. [11] betonen zudem die Notwendigkeit der Einbeziehung von Behandlungsteams, die im Umgang mit dieser Art von Leid geschult sind. In Fällen, in denen psychisches und/oder existenzielles Leid die Hauptindikation für eine PS ist, sind daher **besondere Vorsichtsmaßnahmen**

Besondere Vorsichtsmaßnahmen

zu empfehlen wie ein umfassendes Assessment durch Palliativmediziner und/oder Mitarbeitende mit Expertise in Psychologie, sozialer Arbeit oder Spiritualität. Bei den Patienten sollte dann zunächst eine **intermittierende Sedierung**

Intermittierende Sedierung

durchgeführt werden, da manchmal allein die Unterbrechung einer belastenden Situation zu einer ausreichenden Erleichterung des existenziellen Leids führen kann. Stellt sich die Frage nach der Anwendung einer tiefen und kontinuierlichen Sedierung, wird empfohlen, eine **ethische Beratung**

Ethische Beratung

bzw. ethische Fallbesprechung in Anspruch zu nehmen [4, 5].

Grundsätzlich und unabhängig von der Natur des Leids wird die Konsultation eines **spezialisierten Palliativteams**

Spezialisiertes Palliativteam

oder anderer medizinischer Fachkräfte dringend empfohlen, um die Unbehandelbarkeit des Leids festzustellen.

### Merke

Eine der Voraussetzungen für die Indikationsstellung einer PS ist das Vorliegen von refraktärem Leid [4, 5].

### Merke

Besondere Vorsichtsmaßnahmen werden empfohlen, wenn psychisches und/oder existenzielles Leid die Hauptindikation für eine PS ist [4, 5].

## Entscheidungsprozess

### Ethische Überlegungen

Die Anwendung einer PS muss sorgfältig abgewogen werden. Der Verlust bzw. die Einschränkung der **Interaktionsfähigkeit**

Interaktionsfähigkeit,

die für die meisten Patienten auch in weit fortgeschrittenen Krankheitsstadien ein wichtiger Aspekt der Lebensqualität ist, sollte ausführlich mit dem Patienten und seinen Zugehörigen besprochen werden [14].

Bedenken wegen einer **Lebensverkürzung**

Lebensverkürzung

durch PS sollten ebenfalls thematisiert werden. Gemäß einigen Studien scheint eine tiefe und kontinuierliche PS, wenn sie indiziert ist und richtig eingesetzt wird, keine nachteiligen Auswirkungen auf das Überleben von Patienten mit fortgeschrittener Krebserkrankung zu haben; im Rahmen der Stressreduktion durch die Sedierung wird sie manchmal sogar mit einem längeren Überleben in Verbindung gebracht [15, 16]. Dennoch kann ein geringes individuelles Risiko der Lebensverkürzung nicht völlig ausgeschlossen werden.

Auf der anderen Seite wird vor der Nichtverabreichung einer PS gewarnt, weil dies zu **vergeblichen Therapieversuchen**

Vergebliche Therapieversuche

führen kann, die das Leid des Patienten verlängern und erhöhen [17]. Um den Entscheidungsprozess zu unterstützen, ist es wichtig, zwischen PS und Tötung auf Verlangen zu unterscheiden, und zwar in Bezug auf

- das Ziel (Linderung des Leids und nicht Beendigung des Lebens),
- die Mittel (proportionale Herabsetzung des Bewusstseins bis zur Linderung des Leids und nicht Herbeiführung des Todes durch Verabreichung von Medikamenten in totbringender Dosierung),
- die Ergebnisse (Linderung des Leids mit lebensverkürzender Wirkung als außergewöhnlicher Nebenwirkung und nicht lebensverkürzend per Definition) und
- den Zeitpunkt (kontinuierliche tiefe PS bleibt der terminalen Phase des Lebens vorbehalten) [4].

#### Merke

Ziel der PS ist es, refraktäres Leid zu lindern, und nicht, das Leben zu verkürzen [4, 5].

### Informierte Einwilligung: das Prinzip der Autonomie des Patienten

Der Patient soll in alle Phasen des Entscheidungsprozesses einbezogen werden. Das Thema PS sollte bereits im Vorfeld erwartbarer Krisensituationen angesprochen werden, damit die **therapeutischen Präferenzen**

Therapeutische Präferenzen

des Patienten, solange er noch informierte Entscheidungen treffen kann, erfasst und dokumentiert werden können. Es wird auch empfohlen, mit dem Einverständnis des Patienten die **Angehörigen**

Angehörige

bzw. **Zugehörigen**

Zugehörige

in diese Gespräche einzubeziehen, damit sie die getroffenen Entscheidungen verstehen können und um ihnen gegebenenfalls Zeit zu geben, sich zu verabschieden, aber auch um sie in dieser schwierigen Phase zu unterstützen.

Wenn der Patient nicht entscheidungsfähig ist, sollten die persönlichen Präferenzen aus seiner **Patientenverfügung**

Patientenverfügung

in Betracht gezogen werden. Wenn keine Patientenverfügung vorliegt, sollten zuvor geäußerte Präferenzen berücksichtigt oder **mutmaßliche Behandlungspräferenzen**

Mutmaßliche Behandlungspräferenzen,

wenn möglich, beim gesetzlichen Vertreter oder den Angehörigen erfragt werden.

In der Indikationsstellung ist eine **Kooperation**

Kooperation

zwischen Arzt und Patient erforderlich, um die Refraktärität des Leids zu überprüfen. Dabei übernimmt der Arzt die Prüfung, ob bzw. welche Therapieoptionen noch verbleiben, welcher Zeitrahmen für entsprechende Therapieversuche erforderlich wäre und welche Komplikationen und unerwünschte Wirkungen dabei zu erwarten wären. Dem Patienten obliegt die Prüfung, ob das Leid als unerträglich empfunden wird und welche Therapieversuche aus seiner Perspektive noch zu ertragen wären. Aus diesen beiden Perspektiven kann dann die Indikation der PS gestellt werden. Wenn die Indikation gestellt wurde, soll der Patient über Absicht, Wirkung, geplante Dauer, unerwünschte Wirkungen und Risiken aufgeklärt und seine Zustimmung eingeholt werden.

#### Merke

Der Patient soll in alle Phasen des Entscheidungsprozesses einbezogen werden.

### Form der Sedierung: das Prinzip der Proportionalität

Das Prinzip der Proportionalität besteht darin, dass die **niedrigstmögliche Dosis**

Niedrigstmögliche Dosis

des Medikaments, die zur Linderung des Leids erforderlich ist, gewählt werden sollte [4, 5]. Bei einigen Patienten kann bereits eine leichte bis mäßige Sedierung, bei der sie erweckbar bleiben, eine angemessene Linderung ohne vollständigen Verlust der interaktiven Funktion bewirken (Sedierungstiefe von −1 bis −3 auf der Richmond Agitation Sedation Scale – Palliative Version [**RASS-PAL**

RASS-PAL];

https://www.interiorhealth.ca/sites/default/files/PDFS/826582-richmond-agitation-sedation-scale.pdf; [18]). Eine tiefere Sedierung (RASS-PAL −4 bis −5) sollte in Betracht gezogen werden, wenn sich eine leichte Sedierung als unwirksam erwiesen hat oder wenn klar ist, dass diese nicht rechtzeitig zu einer ausreichenden Linderung führen wird, etwa in Notfällen am Lebensende.

Ebenso sollte die **Dauer**

Dauer

der PS an die individuelle Situation des Patienten angepasst werden. Die PS kann kontinuierlich über einen längeren Zeitraum (mehr als 48 h) und bis zum Tod angewendet werden. Sie kann auch intermittierend (weniger als 48 h) in einem früheren Stadium der Krankheit erfolgen, entweder um dem Patienten vorübergehend Erleichterung zu verschaffen, bis andere Behandlungsmethoden erfolgreich sind, oder um dem Patienten eine Pause von der aktuell belastenden Situation zu ermöglichen. Da hier das Ziel darin besteht, das Bewusstsein nach der Sedierung so weit wie möglich wiederherzustellen, sollte versucht werden, die **physiologische Stabilität**

Physiologische Stabilität

innerhalb der mit dem Patienten vereinbarten therapeutischen Grenzen zu erhalten und die **künstliche Hydratation**

Künstliche Hydratation

im Hinblick auf die voraussichtliche Dauer der Sedierung zu erwägen.

Die Option einer kontinuierlichen tiefen Sedierung sollte erwogen werden, wenn eine intermittierende oder eine kontinuierliche leichte Sedierung nicht ausreicht, um das Leid angemessen zu lindern.

Im Rahmen des SedPall-Projekts wurde der Begriff „vorübergehend“ dem Begriff „intermittierend“ vorgezogen, da letzterer mit einer Wiederholung der Sedierungsphasen assoziiert wird, die nicht zwingend erforderlich ist, zudem wurde statt „kontinuierliche Sedierung“ die Formulierung „sediert bis zum Versterben“ gewählt [5, 10].

#### Merke

Die PS soll proportional sein; die niedrigstmögliche Dosis des Medikaments, die zur Linderung des Leids erforderlich ist, sollte gewählt werden.

### Unabhängiger Entscheidungsprozess bezüglich der Flüssigkeitszufuhr

Die meisten Patienten reduzieren von sich aus ihre Flüssigkeitszufuhr in der letzten Lebensphase. Diese **Flüssigkeitsrestriktion**

Flüssigkeitsrestriktion

kann Symptome wie Müdigkeit, Verstopfung oder Mundtrockenheit verursachen oder verstärken. Andererseits kann sie auch Vorteile haben und durch Sekretreduktion die Symptomlast senken [19]. Daher sollte im Rahmen der PS die Flüssigkeitszufuhr weder automatisch begonnen noch fortgesetzt oder unterbrochen werden. Die Entscheidung über eine **künstliche Flüssigkeitszufuhr**

Künstliche Flüssigkeitszufuhr

sollte von der Entscheidung für die PS getrennt werden. Sie sollte die persönlichen Präferenzen, die Lebensqualität und das psychische und spirituelle Wohlbefinden des Patienten berücksichtigen [20, 21]. In Fällen, in denen es religiöse oder kulturelle Vorbehalte gegen den Abbruch der Flüssigkeitszufuhr gibt, sollte diese fortgesetzt werden, es sei denn, es gibt Hinweise auf einen direkten Schaden für den Patienten durch den Eingriff.

#### Merke

Die Entscheidung über die Anwendung einer PS und die Entscheidung, ob während dieser eine künstliche Flüssigkeitszufuhr erfolgen soll, werden getrennt voneinander getroffen [4, 5].

### Lebenserwartung

Auf der Grundlage der drei Prinzipien (1) Refraktärität des Leids, (2) Proportionalität und (3) unabhängige Entscheidung über die Hydratation wurde kein erforderlicher Wert für die verbleibende Lebenserwartung definiert, wobei die intermittierende PS in einem frühen Stadium der Palliativphase verabreicht werden kann. Die tiefe und kontinuierliche Sedierung sollte der **letzten Lebensphase**

Letzte Lebensphase

vorbehalten bleiben. Es ist jedoch anzumerken, dass während der Überarbeitung des EAPC-Rahmenwerks kein Konsens über die genaue Definition der letzten Lebensphase erzielt wurde.

## Empfohlener stufenweiser pharmakologischer Ansatz

Der stufenweise pharmakologische Ansatz ist in Tab. 1 übersichtlich dargestellt.

**Tab. 1:** Medikation für die palliative Sedierung

Stufe	Medikament	Dosierung	
*1*	Midazolam	Erhaltungsdosis:	Startbolus:
		– 1 mg/h (s.c. und i.v.) – Bei Bedarf alle 1–2 h in Kombination mit einem weiteren Bolus anpassen – Bei Dosen von mehr als 10 mg/h sollte die Hinzufügung von Levomepromazin in Betracht gezogen werden	Leichte Sedierung angestrebt: – 2,5 mg s.c. – 1,25 mg i.v.
			Tiefe Sedierung angestrebt: – 5–10 mg s.c. – 2,5–5 mg i.v.
			– Ein Bolus mit der Hälfte der Startdosis kann bei Bedarf nach 20 min s.c. oder 5 min i.v. wiederholt werden – Die Verabreichung von 2 bis 3 zusätzlichen Boli in den ersten Stunden der palliativen Sedierung kann notwendig sein
*2* *In Kombination mit Midazolam*	Levomepromazin	– Als intermittierender Bolus: 12,5–25 mg alle 6–8 h (s.c./i.v.) – Als kontinuierliche Infusion: 0,5–8 mg/h (s.c./i.v.)	
*3*	Propofol	– Anfangsdosis: 1 mg/kg pro h (i.v.) – Bei Bedarf Erhöhung um 0,5 mg/kg pro h alle 30 min	

### Stufe 1

Ein gut kontrollierbares Benzodiazepin wie **Midazolam**

Midazolam

sollte in der Erstlinientherapie eingesetzt werden (alternativ **Lorazepam**

Lorazepam).

Midazolam hat den Vorteil, dass sein schneller Wirkungseintritt und seine kurze Halbwertszeit eine schnelle Anpassung der Sedierungstiefe ermöglichen. Als seltene Nebenwirkung kann es zu einer paradoxen Agitation kommen [4].

### Stufe 2

Bei Bedarf kann ein **niedrigpotentes Neuroleptikum**

Niedrigpotentes Neuroleptikum

wie **Levomepromazin**

Levomepromazin

in Kombination mit dem Benzodiazepin eingesetzt werden. Das Neuroleptikum hat den Vorteil, dass es schnell sedierend wirkt, eine antipsychotische Wirkung bei Delirium hat und antiemetisch wirkt. Bei refraktärem Delirium, Alkoholismus in der Vorgeschichte und/oder Drogenabhängigkeit kann Levomepromazin als Alternative zu Midazolam in der Erstlinientherapie eingesetzt werden. Unerwünschte Wirkungen können paradoxe Agitation, extrapyramidale Symptome und anticholinerge Wirkungen sein.

### Stufe 3

Bei nicht ausreichender Wirkung der ersten beiden Stufen kann in der dritten Stufe Propofol verwendet werden. Propofol sollte aber von einem Anästhesisten oder einer Person mit ausreichender Erfahrung in der Anwendung und nur bei stationär betreuten Patienten verabreicht werden.

#### Cave

Opioide können zur Linderung von Schmerzen oder Atemnot erforderlich sein, und Neuroleptika wie Haloperidol können zur Behandlung eines Deliriums angezeigt sein. Diese Medikamente können dann beibehalten oder neu angesetzt werden, um die Symptome zu lindern, sollten aber nicht zur Einleitung oder Aufrechterhaltung einer PS angewendet werden.

#### Merke

Ein gut kontrollierbares Benzodiazepin wie Midazolam sollte in der Erstlinientherapie eingesetzt werden.

## Monitoring

Die PS sollte von einem **Arzt**

Arzt

und einer **Pflegekraft**

Pflegekraft

eingeleitet und alle 20 min überwacht werden, bis das gewünschte Maß an Komfort erreicht ist, danach mindestens 3‑mal täglich, wenn eine kontinuierliche Sedierung erfolgt [4]. Bei der Überwachung müssen

- der Schweregrad des Leids (wichtigstes Kriterium),
- das Bewusstseinsniveau sowie
- die mit der PS verbundenen unerwünschten Wirkungen (Delirium, Agitation) und Komplikationen (Aspiration)

berücksichtigt werden.

Die Medikamentendosierung sollte schrittweise angepasst werden, um eine optimale Linderung bei **geringstmöglicher Bewusstseinsunterdrückung**

Geringstmögliche Bewusstseinsunterdrückung

zu erreichen. Wenn eine leichte oder intermittierende Sedierung angestrebt wird, sollte die physiologische Stabilität innerhalb der mit dem Patienten vereinbarten therapeutischen Grenzen aufrechterhalten werden. Der Grad der Sedierung und die **Vitalparameter**

Vitalparameter,

unter anderem Herzfrequenz, Blutdruck und Sauerstoffsättigung, sollten regelmäßig überwacht werden. Bei der tiefen kontinuierlichen Sedierung in der letzten Lebensphase sollten nur die Parameter beobachtet werden, die therapeutische Konsequenzen erfordern würden. Das Behandlungsteam muss das gleiche Maß an persönlicher Betreuung aufrechterhalten wie vor der PS.

### Merke

Die Medikamentendosierung sollte schrittweise angepasst werden, um eine optimale Linderung bei geringstmöglicher Unterdrückung des Bewusstseins zu erreichen [4, 5].

## Begleitung der Angehörigen

Es wird empfohlen, den Angehörigen **besondere Aufmerksamkeit**

Besondere Aufmerksamkeit

zu widmen. Sie sollten mit dem Einverständnis des Patienten in den Entscheidungsprozess einbezogen und während des gesamten Verfahrens regelmäßig informiert werden. Wenn möglich, sollte ihnen die Gelegenheit gegeben werden, sich vor Beginn der Sedierung vom Patienten zu verabschieden. Wichtig ist auch, dass sie während der Sedierung und nach dem Tod des Patienten begleitet werden.

### Merke

Die Angehörigen müssen vor, während und nach der Sedierung begleitet werden.

## Begleitung des behandelnden Teams

Für das behandelnde Team kann die PS eines Patienten belastend sein. Dies gilt insbesondere, wenn Uneinigkeit über die Angemessenheit des Eingriffs besteht und wenn sich der Prozess in die Länge zieht. Daher ist es wichtig, dass jedes Mitglied des Teams die medizinische Begründung und die Ziele der PS versteht. Wann immer es möglich ist, sollte dies in Teamsitzungen oder Fallkonferenzen angesprochen werden, sowohl vor als auch nach dem Ereignis, um die fachlichen und emotionalen Fragen zu besprechen, die mit solchen Entscheidungen verbunden sind.

### Merke

Es ist wichtig, dass jedes Mitglied des Behandlungsteams die medizinische Begründung für die PS und die Ziele der Behandlung versteht. Teamsitzungen oder Fallkonferenzen können hierbei eine nützliche Ressource sein.

## Fazit für die Praxis

- Die palliative Sedierung (PS) ist eine Ultima Ratio zur Behandlung von refraktärem Leid.
- Die Bestimmung der Refraktärität erfolgt gemeinsam durch den Patienten und ein multiprofessionelles und interdisziplinäres Behandlungsteam.
- Besondere Vorsichtsmaßnahmen werden empfohlen, wenn psychisches und/oder existenzielles Leid die Hauptindikation für eine PS ist.
- Form und Dauer der PS sollen an die individuelle Situation des Patienten angepasst werden.
- Besondere Aufmerksamkeit ist den Angehörigen und dem Behandlungsteam vor, während und nach der Sedierung zu widmen.

## CME-Fragebogen

Worauf zielt die palliative Sedierung?

Auf die Linderung von Leid, das der Patient als unerträglich empfindet

Auf die Beschleunigung des Todes bei Patienten mit refraktärem Leid und lebenslimitierenden Erkrankungen

Auf die Linderung von refraktärem Leid durch die überwachte proportionale Bewusstseinsreduktion bei Patienten mit lebenslimitierenden Erkrankungen

Auf die kontinuierliche und tiefe Bewusstseinsreduktion bei Patienten mit refraktärem Leid

Auf die Linderung von refraktärem Leiden, wenn der Patient am Lebensende ist

Was sollten Sie bei der Indikationsstellung zur palliativen Sedierung beachten?

Ein Leid gilt dann als refraktär, wenn Ärzte alle konventionellen Behandlungsmethoden ausgeschöpft haben.

Eine palliative Sedierung kann nur bei refraktären körperlichen Symptomen in Betracht gezogen werden, da die Bestimmung der Refraktärität von psychischem oder existenziellem Leid nicht möglich ist.

In 45 % der Fälle bezieht sich die Indikation nicht auf ein Symptom, sondern auf ein Cluster von Symptomen.

Existenzielles Leid tritt am Ende des Lebens häufig allein auf.

Die Unbehandelbarkeit des Leids obliegt einer medizinischen Abwägung und die Bestimmung der Refraktärität des Leids erfolgt gemeinsam durch den behandelnden Arzt und den Patienten.

Eine 65-jährige Patientin mit metastasiertem Ovarialkarzinom und Ileusproblematik mit massiver Übelkeit und Erbrechen erklärt Ihnen, dass sie nicht mehr so weiterleben möchte, und bittet um eine palliative Sedierung. Was tun Sie als behandelnder Arzt?

Ich erkläre der Patientin, dass dies nicht möglich ist, da die palliative Sedierung Patienten vorbehalten ist, deren Prognose in Tagen oder Stunden berechnet wird.

Ich leite eine kontinuierliche, tiefe Sedierung ein, damit die Patientin nicht mehr leiden muss.

Ich überprüfe die durchgeführte Therapie und schaue, ob sie verbessert werden kann. Ich ziehe eventuell das Palliativteam oder andere Experten hinzu, um die Refraktärität der Symptome zu bestimmen.

Ich ziehe einen Psychiater beratend hinzu und verschreibe ein Antidepressivum, da die Patientin wahrscheinlich an einer Depression leidet.

Ich erkläre der Patientin, dass dies nicht möglich ist, da aktive Sterbehilfe nicht erlaubt ist.

Auf welchen Punkt sollten Sie bei dem Entscheidungsprozess über eine palliative Sedierung bei entscheidungsfähigen Patienten achten?

Das Thema der palliativen Sedierung sollte bei Patienten im Vorfeld von Krisensituationen besprochen werden, damit diese ihre therapeutischen Präferenzen äußern können.

Der Verlust der Fähigkeit, mit Angehörigen und Behandlungsteam zu interagieren, spielt für Patienten am Lebensende eine untergeordnete Rolle.

Um sie nicht zu belasten, sollten Angehörige erst dann in den Entscheidungsprozess einbezogen werden, wenn der Patient selbst nicht in der Lage ist, Entscheidungen zu treffen.

Bei ethischen Konflikten sollte keine palliative Sedierung eingeleitet werden.

Die Zustimmung des gesetzlichen Vertreters und/oder der Angehörigen ist in jedem Fall erforderlich, bevor eine Sedierung eingeleitet wird.

Auf welchen Punkt sollten Sie bei dem Entscheidungsprozess zur Flüssigkeitszufuhr achten?

Bei Beginn einer palliativen Sedierung sollte die künstliche Flüssigkeitszufuhr systematisch unterbrochen oder gar nicht erst begonnen werden, damit es nicht zu einer Zunahme der Symptomlast durch eine Erhöhung der Sekretion kommt.

Eine künstliche Flüssigkeitszufuhr von 1000 ml/Tag sollte in erster Linie bei einer palliativen Sedierung erfolgen, um Mundtrockenheit und Verstopfung nicht zu verstärken.

Die Entscheidung über die Flüssigkeitszufuhr ist eine medizinische Entscheidung, bei der religiöse oder kulturelle Faktoren des Patienten nicht berücksichtigt werden sollten.

Die Anwendung einer palliativen Sedierung und die Entscheidung, ob währenddessen eine künstliche Flüssigkeitszufuhr erfolgen soll, sollten in zwei unterschiedliche Entscheidungsprozesse getrennt werden.

Eine Entscheidung über künstliche Flüssigkeitszufuhr während der Sedierung muss nicht getroffen werden, da die Patienten am Lebensende ihre Flüssigkeitszufuhr von selbst reduzieren.

Welchen Aspekt sollten Sie bei der Wahl der Form der palliativen Sedierung berücksichtigen?

Die Wahl der Form der palliativen Sedierung bei isoliertem refraktärem existenziellem Leiden unterliegt denselben Bedingungen wie bei refraktären körperlichen Symptomen.

Eine tiefe palliative Sedierung sollte sofort eingeleitet werden, um das Leiden des Patienten nicht zu verlängern.

Das Prinzip der Proportionalität bedeutet, dass die Dosierung des Medikaments, das zur Linderung des Leidens erforderlich ist, proportional zur Lebenserwartung des Patienten gewählt werden soll.

Die intermittierende palliative Sedierung bleibt der terminalen Phase des Lebens vorbehalten.

Eine tiefe Sedierung sollte in Betracht gezogen werden, wenn sich eine leichte Sedierung als unwirksam erwiesen hat oder wenn klar ist, dass diese nicht rechtzeitig zu einer ausreichenden Linderung führen wird.

Ein 72-jähriger Patient mit fortgeschrittenem Hypopharynxkarzinom leidet an einer akuten Asphyxie aufgrund einer massiven Obstruktion der Atemwege. Diese Situation wurde schon vor einiger Zeit mit ihm besprochen und der Patient hat seinen Wunsch nach einer palliativen Sedierung mitgeteilt. Dieser Wunsch wurde auch in seiner Patientenverfügung formuliert. Was tun Sie als behandelnder Arzt?

Da der Patient bereits Opioide gegen seine Atemnot erhält, erhöhe ich die Dosis, um eine Sedierung des Patienten herbeizuführen.

Ich verlege den Patienten umgehend auf die Intensivstation.

Ich beginne eine palliative Sedierung mit 1 mg Midazolam pro Stunde in Kombination mit einem initialen Bolus von 2,5 mg i.v. und plane, die Situation in 20 min erneut zu evaluieren.

Ich organisiere eine Fallbesprechung für den nächsten Tag. Solche Entscheidungen sollten nicht in Eile getroffen werden.

Ich beginne eine palliative Sedierung mit 5 mg Midazolam pro Stunde in Kombination mit einem initialen Bolus von 5 mg i.v., setze die Opioide ab und plane, die Situation in 20 min erneut zu evaluieren.

Welcher medikamentöse Ansatz ist bei der Einleitung einer palliativen Sedierung in erster Linie zu bevorzugen?

Ein Anästhetikum wie Propofol aufgrund seines schnellen Wirkungseintritts und seiner kurzen Plasmahalbwertszeit

Ein niedrigpotentes Neuroleptikum wie Levomepromazin

Ein schnell wirkendes Benzodiazepin mit kurzer Plasmahalbwertszeit wie Midazolam

Opioide, die den Vorteil haben, Schmerzen und Atemnot zu lindern und eine angemessene Sedierung herbeizuführen

Ein hochpotentes Neuroleptikum wie Haloperidol bei refraktärem Delirium, Alkoholismus in der Vorgeschichte und Drogenabhängigkeit

Sie haben eine tiefe kontinuierliche palliative Sedierung bei einem 84-jährigen Patienten eingeleitet, der an refraktären Schmerzen bei metastasiertem Bauchspeicheldrüsenkrebs leidet und dessen Lebenserwartung auf wenige Stunden bis Tage geschätzt wird. Wie überwachen Sie den Patienten?

Ich überwache die vitalen physiologischen Parameter wie Herzfrequenz, Blutdruck und Sauerstoffsättigung dreimal täglich.

Ich passe schrittweise die Medikamentendosen an, um eine optimale Linderung bei geringstmöglicher Unterdrückung des Bewusstseins zu erreichen.

Ich stelle jegliche Pflege ein und lasse den Patienten in Ruhe.

Ich setzte die bisherige Medikation mit Opioiden ab.

Ich passe die Medikamentendosen schrittweise an das Bewusstseinsniveau des Patienten an.

Ein 58-jähriger Patient mit Glioblastom wurde auf Ihrer Station aufgenommen, weil der fortschreitende Verlust seiner Mobilität und damit seiner Autonomie für ihn unerträglich ist. Er wünscht sich eine palliative Sedierung. Seine Lebenserwartung wird auf zwei bis drei Monate geschätzt. Welche Aspekte berücksichtigen Sie bei Ihrer Entscheidung?

Ich berücksichtige, dass die Angehörigen mit der palliativen Sedierung einverstanden sind.

Ich berücksichtige, dass das Team das Leid des Patienten nicht mehr ertragen kann.

Ich berücksichtige, dass zusammen mit dem Patienten die Refraktärität des Leids festgestellt wird.

Ich berücksichtige, dass vor Beginn der palliativen Sedierung ein Zeitraum von vier Wochen eingehalten wird, um sicherzustellen, dass der Wunsch des Patienten konstant bleibt.

Ich berücksichtige, dass die Lebenserwartung des Patienten vor dem Beginn einer palliativen Sedierung noch zu lang ist.
